# Early response monitoring of neoadjuvant chemotherapy using [^18^F]FDG PET can predict the clinical outcome of extremity osteosarcoma

**DOI:** 10.1186/s13550-019-0588-4

**Published:** 2020-01-03

**Authors:** Inki Lee, Byung Hyun Byun, Ilhan Lim, Byung Il Kim, Chang Woon Choi, Jae-Soo Koh, Won Seok Song, Wan Hyeong Cho, Chang-Bae Kong, Sang Moo Lim

**Affiliations:** 1 Department of Nuclear Medicine, Korea Cancer Center Hospital, Korea Institute of Radiological and Medical Sciences, Seoul, Korea; 2 Department of Pathology, Korea Cancer Center Hospital, Korea Institute of Radiological and Medical Sciences, Seoul, Korea; 3 Department of Orthopedic Surgery, Korea Cancer Center Hospital, Korea Institute of Radiological and Medical Sciences, Seoul, Korea

**Keywords:** Osteosarcoma, [^18^F]Fluorodeoxyglucose, Positron emission tomography, Standardized uptake value, Event-free survival

## Abstract

**Background:**

To propose a personalized therapeutic approach in osteosarcoma treatment, we assessed whether sequential [^18^F]FDG PET/CT (PET/CT) could predict the outcome of patients with osteosarcoma of the extremities after one cycle and two cycles of neoadjuvant chemotherapy.

**Methods:**

A total of 73 patients with AJCC stage II extremity osteosarcoma treated with 2 cycles of neoadjuvant chemotherapy, surgery, and adjuvant chemotherapy were retrospectively analyzed in this study. All patients underwent PET/CT before (PET0), after 1 cycle (PET1), and after the completion of neoadjuvant chemotherapy (PET2), respectively. Maximum standardized uptake value (SUV_max_) (corrected for body weight) and the % changes of SUV_max_ were calculated, and histological responses were evaluated after surgery. Receiver-operating characteristic (ROC) curve analyses and the Cox proportional hazards models were used to analyze whether imaging and clinicopathologic parameters could predict event-free survival (EFS).

**Results:**

A total of 36 patients (49.3%) exhibited a poor histologic response and 17 patients (23.3%) showed events (metastasis in 15 and local recurrence in 2). SUV_max_ on PET2 (SUV2), the percentage change of SUV_max_ between PET0 and PET1 (Δ%SUV01), and between PET0 and PET2 (Δ%SUV02) most accurately predicted events using the ROC curve analysis. SUV2 (relative risk, 8.86; 95% CI, 2.25–34.93), Δ%SUV01 (relative risk, 5.97; 95% CI, 1.47–24.25), and Δ%SUV02 (relative risk, 6.00; 95% CI, 1.16–30.91) were independent predicting factors for EFS with multivariate analysis. Patients with SUV2 over 5.9 or Δ%SUV01 over − 39.8% or Δ%SUV02 over − 54.1% showed worse EFS rates than others (*p* < 0.05).

**Conclusions:**

PET evaluation after 1 cycle of presurgical chemotherapy can predict the clinical outcome of extremity osteosarcoma. [^18^F]FDG PET, which shows a potential role in the early evaluation of the modification of timing of local control, can be a useful modality for early response monitoring of neoadjuvant chemotherapy.

## Background

Osteosarcoma is the most common bone tumor that occurs in children and adolescents, although the overall prevalence is not high. The survival rate of osteosarcoma remained at around 20% before the 1980s, but the 5-year event-free survival (EFS) rate increased to 55–75% after neoadjuvant chemotherapy (NAC), and surgical resection became the standard therapy [1]. Despite significant improvements in survival since the introduction of NAC, treatment and outcome have not changed in several decades [2]. According to the National Comprehensive Cancer Network (NCCN) guidelines for bone cancers, all osteosarcoma patients should undergo NAC even in the era of precision medicine [3].

Fluorine 18-fluorodeoxyglucose ([^18^F]FDG) positron emission tomography (PET) has been used to evaluate the prognosis of many cancers [4]. Many groups reported that baseline and post-chemotherapy PET could predict the prognosis of osteosarcoma [5]. However, few have focused on the early response to NAC with PET [6]. Therefore, it is necessary to evaluate the definite prognostic factors with [^18^F]FDG PET. Furthermore, it is also important to determine at which time point during treatment should [^18^F]FDG PET be used for the best optimal prognosis.

The parameters available for [^18^F]FDG PET are diverse, which include SUV_max_, mean SUV (SUV_mean_), peak SUV (SUV_peak_), metabolic tumor volume (MTV), and total lesion glycolysis (TLG). Furthermore, various texture features are also available for analysis. Among many PET parameters, SUV_max_ is the most widely used parameter. Although it can be affected by ambient noise because it is a value from a single voxel, it is easily measurable and is used as a representative value for tumors [7]. We previously investigated how SUV_max_ can predict histologic response in osteosarcoma [8]. We also found that tumor necrosis at the point of maximum metabolic activity was significantly correlated with the histologic response of the entire resected specimen [8]. It suggests that the maximally metabolically active portion of the tumor is the most aggressive portion and this single pixel represents the aggressiveness of cancer. Therefore, the SUV_max_, which is easily measurable and universally used, was selected for predicting the prognosis of osteosarcoma in this study.

The overall survival of extremity osteosarcoma patients has plateaued in several decades and this plateau of clinical outcome highlights the need for a novel therapeutic approach in osteosarcoma treatment [2]. In the present study, we assessed whether sequential [^18^F]FDG positron emission tomography/computed tomography (PET/CT) could predict the outcome of patients with osteosarcoma of the extremities after 1 cycle and 2 cycles of NAC.

## Patients and methods

### Patients

From June 2007 to September 2013, patients who were initially diagnosed with osteosarcoma histologically were retrospectively analyzed. Among them, patients were selected based on the following criteria: (1) NAC, (2) surgical resection, (3) postoperative chemotherapy, (4) [^18^F]FDG PET/CT performed before, 1 cycle after, and 2 cycles after NAC, (4) available follow-up data for at least 2 years to determine EFS. Patients with the American Joint Committee on Cancer (AJCC) stage I, III, or IV at initial presentation were excluded from the current study. Due to a lower likelihood of metastases in patients with AJCC stage I, these patients were treated with surgery alone [9]. It was reported that patients with AJCC stage III or IV showed poor outcomes. Therefore, these subjects were excluded [9].

Patients over 40 years of age or with non-extremity osteosarcoma were also excluded. As a result, a total of 73 patients were retrospectively analyzed in the present study. The study design and exemption of informed consents were approved by the Institutional Review Board of the Korea Cancer Center Hospital (IRB no. KIRAMS 2019-03-009).

All subjects underwent 2 cycles of NAC with high-dose methotrexate, adriamycin, and cisplatin according to the modified T10 protocol. The intervals between the end of the first cycle of chemotherapy and initiation of the second cycle, and between the end of the second cycle and surgery, were around 3 weeks.

Subjects who did not show any local recurrence or metastasis were censored at the last follow-up date, with a cutoff date of May 29, 2017. The length of follow-up ranged from 13 to 117 months (median, 83 months). Evaluation of local recurrence or metastasis was confirmed by histological diagnosis or follow-up imaging including ultrasonography, X-ray, CT, magnetic resonance imaging (MRI), bone scan, and [^18^F]FDG PET/CT for at least 6 months. After NAC, the histological response was evaluated with post-surgical specimens, of which necrosis over 90% was considered a good response [10]. Otherwise, the histological response was regarded as a poor response.

### [^18^F]FDG PET/CT image acquisition

After fasting for at least 6 h, children under 15 years old were injected intravenously with 7.4 MBq/kg of [^18^F]FDG and children over 15 years old or adults were injected with 370 MBq of [^18^F]FDG. The patients’ blood glucose level was evaluated before administration of [^18^F]FDG and the value did not exceed 7.2 mmol/L. PET images were obtained 1 h after an injection of [^18^F]FDG using a PET/CT scanner (Biograph6; Siemens Medical Solutions, Malvern, PA). CT images were obtained for attenuation correction before the acquisition of PET images (120 kVp, 30 mA, 0.6 s/CT rotation, and a pitch of 6). PET images were reconstructed using an ordered subset expectation maximization algorithm (iteration 2 and subset 8). Effective dose from FDG PET was 1.9 × 10^−2^ mSv/MBq and effective dose from low dose attenuation CT was about 4 mSv. Each uptake time before imaging at baseline [^18^F]FDG PET (PET0) before NAC, interim [^18^F]FDG PET (PET1) after 1 cycle of NAC, and post-chemotherapy [^18^F]FDG PET (PET2) was 57.1 ± 2.7 min, 57.3 ± 2.5 min, and 55.9 ± 3.1 min, respectively.

### Imaging analysis

The maximum standardized uptake value (SUV_max_) of the primary tumor was evaluated using the Syngo software (Siemens Medical Systems, Iselin, NJ). A volume of interest covering the primary tumor was manually drawn to evaluate SUV_max_. The SUV_max_ of PET0 before NAC was defined as SUV0, the SUV_max_ for PET1 after 1 cycle of NAC was defined as SUV1, and the SUV_max_ of PET2 was defined as SUV2. The percentage change in SUV_max_ between SUV0 and SUV1 (Δ%SUV01) was defined as follows: Δ%SUV01 = (SUV1–SUV0)/SUV0 × 100. The percentage change in SUV_max_ between SUV0 and SUV2 (Δ%SUV02) and between SUV1 and SUV2 (Δ%SUV12) was also defined by the same formula.

### Statistical analysis

To predict events, the receiver operating characteristic (ROC) curve analyses were done with the parameters of [^18^F]FDG PET images. The areas under the curve (AUCs) were calculated and the optimal cut-off values for each parameter were evaluated based on the Youden index. The association between SUV parameters and outcome (EFS) was evaluated using univariate and multivariate Cox regression analysis. The univariate and multivariate Cox proportional hazards model with a backward conditional stepwise procedure was used to evaluate prognostic variables, including SUV, age, gender, AJCC stage, tumor location, histologic subtype, and histologic response. We confirmed the proportional hazards assumption using Schoenfeld’s test and plotted the martingale residuals against continuous variables to detect nonlinearity. For the multivariate Cox proportional hazards model, the clinicopathological parameters and each PET parameters were taken into the analyses and three separate analyses with all the clinicopathological parameters were performed as follows: (1) using SUV2; (2) using Δ%SUV01; (3) using Δ%SUV02. For the Cox regression analysis, all variables were evaluated after dichotomization. EFS curves were estimated using the Kaplan-Meier methods, and differences in survival between groups were assessed by the log-rank test.

Statistical tests were performed using MedCalc (version 12.3; MedCalc Software, Ostend, Belgium). All *p* values were 2-sided, and *p* values of < 0.05 were accepted as indicating statistical significance.

## Results

### Clinical characteristics of patients

A total of 73 patients (53 males and 20 females; mean age, 17 years; range, 8–38 years) were analyzed in this study. PET0 was performed 2.8 ± 2.8 days before the initiation of NAC. PET1 and PET2 were done 15.1 ± 5.2 days and 16.2 ± 4.3 days after the end of cycle one and cycle two of NAC, respectively. Then, surgery was performed 5.2 ± 4.5 days after PET2. About half of the patients (51%) exhibited a good histologic response in the resected specimens after NAC (Table 1).

**Table 1:** Patient characteristics

Characteristics	No. of patients (%), *n* = 73
Age, years	
≤ 15	44 (60%)
> 15	29 (40%)
Sex	
Male	53 (73%)
Female	20 (27%)
AJCC stage	
IIA	31 (43%)
IIB	42 (57%)
Location	
Femur	37 (51%)
Tibia	28 (38%)
Others	8 (11%)
Pathologic subtype	
Osteoblastic	57 (78%)
Chondroblastic	6 (8%)
Fibroblastic	7 (10%)
Other	3 (4%)
Histologic response	
Good	37 (51%)
Poor	36 (49%)
Total	73 (100%)

Seventeen patients (23%) had experienced events (distant metastasis in 15 patients and local recurrence in 2 patients). Metastases were located in the lung in 12 patients and in the bone in 3 patients. Among the 17 patients who experienced events, 3 patients died of the disease and there were no cases of non-cancer-related death in the current study. The event-free interval ranged from 5 to 128 months, with a median event-free interval of 95 months. The estimated 2-year and 5-year EFS rates were 82% (95% confidence interval [CI], 78–87%) and 78% (95% confidence interval [CI], 73–83%), respectively.

### Evaluation of PET parameters for predicting prognosis

Each SUV_max_ of the primary tumor at baseline, during NAC, and after NAC are detailed in Table 2. For all patients including event and non-event group, SUV_max_ decreased significantly after cycle one and cycle two of NAC.

**Table 2:** SUV_max_ at before, during, and after neoadjuvant chemotherapy depending on the event

	Parameter	Mean ± SD	*p* value
All patients	SUV0	9.1 ± 5.1	
	SUV1	5.9 ± 3.4	< 0.001^*,‡^
	SUV2	4.9 ± 3.2	< 0.001^†,‡^
Non-event group	SUV0	9.2 ± 5.4	
	SUV1	5.7 ± 3.4	< 0.001^*,‡^
	SUV2	4.6 ± 3.4	< 0.001^†,‡^
Event group	SUV0	8.7 ± 4.1	
	SUV1	6.8 ± 2.3	0.017^*,‡^
	SUV2	5.9 ± 2.5	0.003^†,‡^

*SUV0* SUV_max_ of baseline [^18^F]FDG PET before neoadjuvant chemotherapy, *SUV1* SUV_max_ for interim [^18^F]FDG PET after 1 cycle of chemotherapy, *SUV2* SUV_max_ of postchemotherapy [^18^F]FDG PET, *SD* standard deviation

^*^p value between SUV0 and SUV1

^†^*p* value between SUV0 and SUV2

^‡^Statistically significant with *p* value < 0.05

**Fig. 1:**
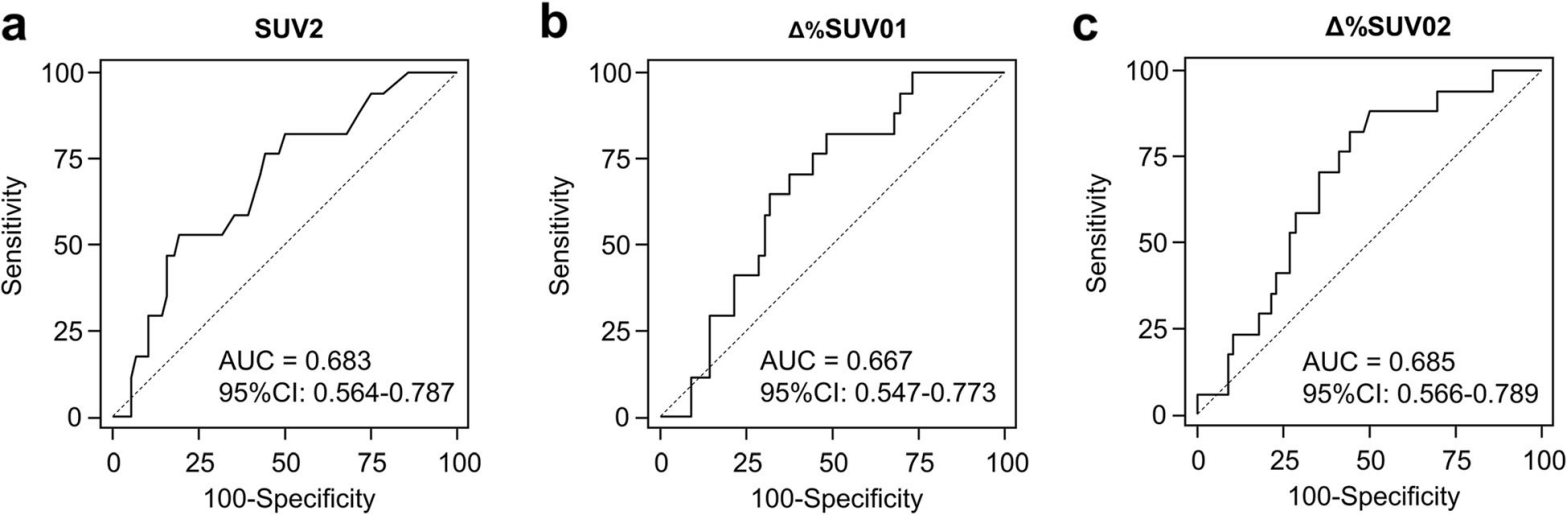
ROC curves of SUV2, Δ%SUV01, and Δ%SUV02 using [^18^F]FDG PET for predicting event-free survival of osteosarcoma. SUV2 is the SUV_max_ of the primary tumor after completion of neoadjuvant chemotherapy (**a**). Δ%SUV01 is the percentage change in SUV_max_ between baseline and interim of [^18^F]FDG PET (**b**). Δ%SUV02 is the percentage change in SUV_max_ between baseline and posttherapy of [^18^F]FDG PET/CT (**c**)

ROC curve analyses were performed for predicting events with each PET parameters (Fig. 1). In the ROC curve analyses, SUV2, Δ%SUV01, and Δ%SUV02 could predict events efficiently. Other parameters including SUV0, SUV1, and Δ%SUV12 did not show statistical significance. Based on the Youden index of ROC curve analysis, each optimal cut-off value of SUV2, Δ%SUV01, and Δ%SUV02 was evaluated as 5.9, − 39.8%, and − 54.1%, respectively. With these cut-off points, the sensitivities were 53%, 82%, and 88%, respectively, and the specificities were 80%, 52%, and 50%, respectively.

### Survival analysis

On the univariate analysis, the parameters of histologic response, SUV2, Δ%SUV01, and Δ%SUV02 were significantly correlated with EFS (Table 3). Other clinicopathological variables such as age, gender, AJCC stage, location, and subtype of the tumor were not associated with EFS. As each PET parameter in this study showed significant correlation with each other, separated multiple analysis models were tested. For the multivariate Cox proportional hazards model, clinical parameters and each PET parameters were taken into the analyses. The results showed that SUV2, Δ%SUV01, and Δ%SUV02 were independent predicting factors for EFS (Table 4). In the multivariate model including SUV2 (model 1), SUV2 greater than or equal to 5.9 and poor histologic response were the independent predicting factors for EFS. In the multivariate model 2 (Δ%SUV01), Δ%SUV01 greater than or equal to − 39.8% and poor histologic response were the independent predicting factors for EFS. Δ%SUV02 greater than or equal to − 54.1% were the independent factors for predicting EFS in the multivariate model 3. In model 3, histologic response did not show statistical significance. The regression analysis showed strong association between Δ%SUV02 and histologic response (*p* = 0.007). Due to this dependence, Δ%SUV02 was the only one independent prognostic factor in model 3. The assumption of proportional hazards was not violated for model 1, model 2, or model 3 and there was no significant nonlinearity in these models.

**Table 3:** Univariate Cox proportional hazards models for event-free survival

Parameters	Univariate		
	RR	95% CI	*p* value
Age			
> 15	0.78	0.25–2.42	0.670
Sex			
male	0.61	0.19–1.96	0.407
AJCC stage (vs 2A)			
2B	1.48	0.48–4.56	0.496
Location (vs femur)			
Tibia	0.74	0.23–2.34	0.605
Others	0.68	0.07–6.79	0.739
Pathologic subtype (vs osteoblastic)			
Fibroblastic	<0.01	N/A	0.998
Chondroblastic	1.54	0.25–9.30	0.641
Others	1.54	0.13–18.25	0.734
Histologic response			
Poor	2.97	1.04–8.45	0.041^*^
SUV0			
< 6.2	1.81	0.52–6.28	0.353
SUV1			
**≥** 4.6	3.76	0.97–14.57	0.055
SUV2			
**≥** 5.9	3.89	1.49–10.15	0.005^*^
Δ%SUV01			
**≥**− 39.8%	4.23	1.21–14.78	0.024^*^
Δ%SUV02			
**≥**− 54.1%	6.39	1.45–28.10	0.014^*^

*N/A* not assessed, *Δ%SUV01* the percentage change between SUV0 and SUV1, *Δ%SUV02* the percentage change between SUV0 and SUV2

^*^Statistically significant with *p* value < 0.05

**Table 4:** Multivariate Cox proportional hazards models for event-free survival

Characteristics	N (%)	Multivariate		
		RR	95% CI	*p* value
Model 1 (SUV2)				
Histologic response				
Good	37 (51)	1		
Poor	36 (49)	3.74	1.03–13.65	0.046^*^
SUV2				
< 5.9	32 (44)	1		
≥ 5.9	41 (56)	8.86	2.25–34.93	0.002^*^
Model 2 (Δ%SUV01)				
Histologic response				
Good	37 (51)	1		
Poor	36 (49)	4.81	1.42–16.31	0.012^*^
Δ%SUV01				
<− 39.8%	30 (41)	1		
≥− 39.8%	43 (59)	5.97	1.47–24.25	0.012^*^
Model 3 (Δ%SUV02*)*				
Histologic response				
Good	37 (51)	1		
Poor	36 (49)	2.71	0.75–9.85	0.129
Δ%SUV02				
<− 54.1%	30 (41)	1		
≥− 54.1%	43 (59)	6.00	1.16–30.91	0.032^*^

^*^Statistically significant with *p* value < 0.05

**Fig. 2:**
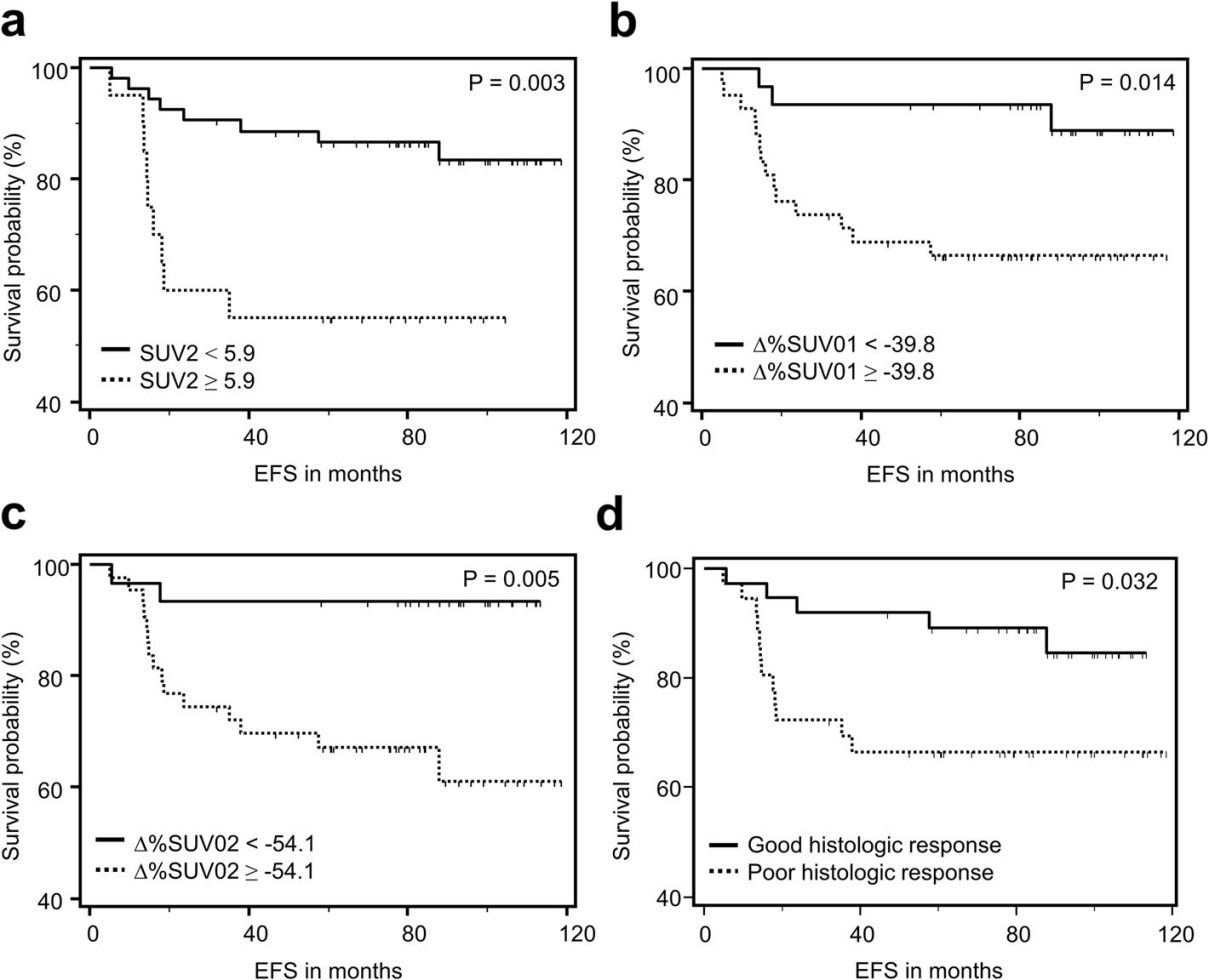
Kaplan-Meier survival curves of the events-free survival depending on the [^18^F]FDG PET parameters including SUV2 (**a**), Δ%SUV01 (**b**), Δ%SUV02 (**c**), and histologic response (**d**). SUV2 is the SUV_max_ of the primary tumor after completion of neoadjuvant chemotherapy. Δ%SUV01 is the percentage change in SUV_max_ between baseline and interim of [^18^F]FDG PET. Δ%SUV02 is the percentage change in SUV_max_ between baseline and post-therapy of [^18^F]FDG PET/CT

Based on the Kaplan-Meier survival analysis (Fig. 2), patients with SUV2 greater than or equal to 5.9 had significantly lower 2-year (60% vs. 91%) and 5-year (55% vs. 87%) EFS rates than patients with SUV2 less than 5.9 (*p* = 0.003). Patients with Δ%SUV01 greater than or equal to − 39.8% showed lower 2-year (74% vs. 94%) and 5-year (66% vs. 94%) EFS rates than patients with Δ%SUV01 less than − 39.8% (*p* = 0.014). Patients with Δ%SUV02 greater than or equal to − 54.1% showed lower 2-year (74% vs. 93%) and 5-year (67% vs. 93%) EFS than patients with Δ%SUV02 less than − 54.1% (*p* = 0.005). Patients with a poor histologic response showed lower EFS than patients with a good histologic response (*p* = 0.032).

**Fig. 3:**
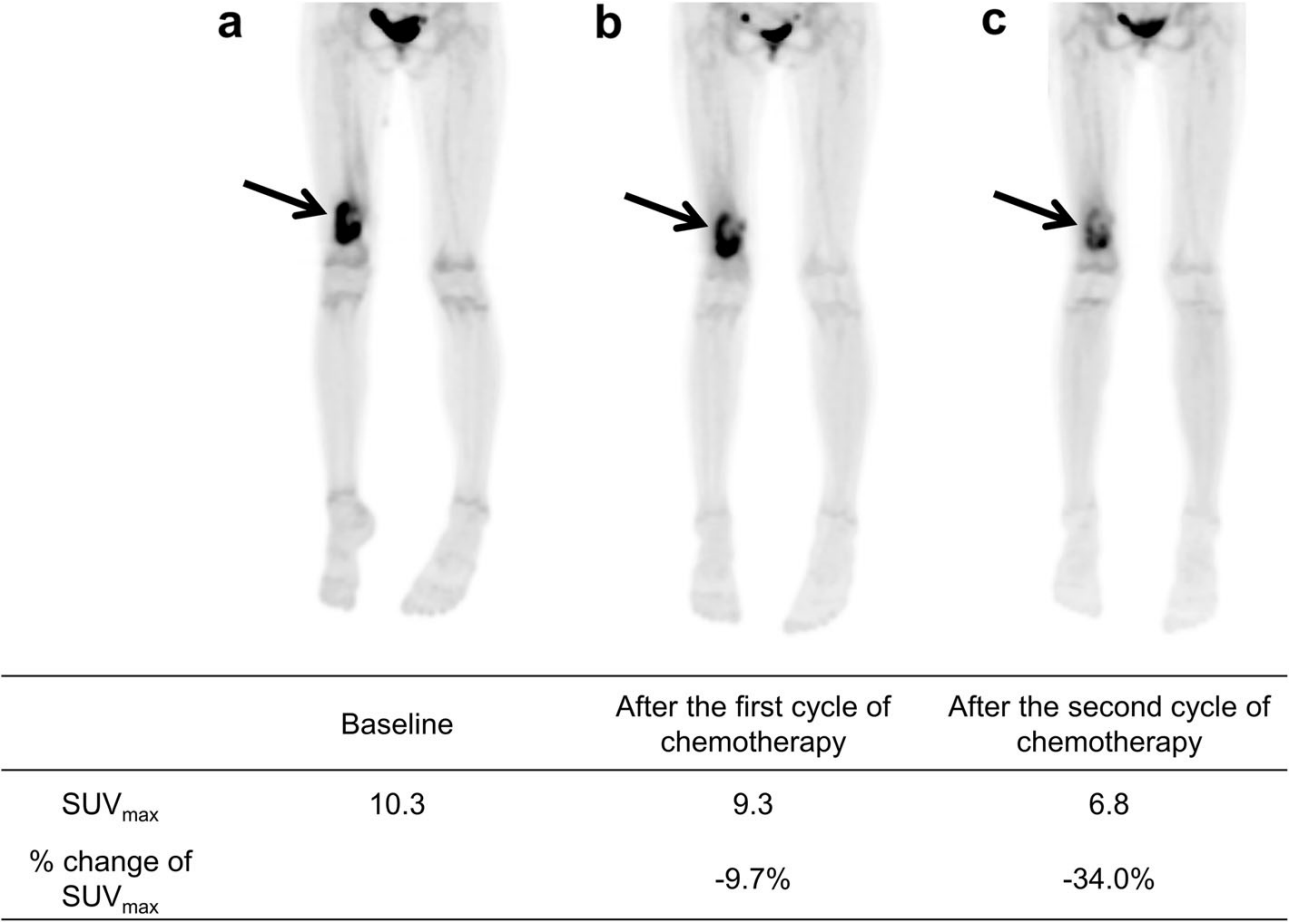
Maximum intensity projection images of [^18^F]FDG PET on the baseline (**a**), after the first cycle of neoadjuvant chemotherapy (**b**), and after the second cycles of neoadjuvant chemotherapy (**c**) of an 11-year-old female with osteosarcoma of the right femur. After neoadjuvant chemotherapy, SUV_max_ has reduced only by 9.7% and 34.0% after 1 cycle and 2 cycles of neoadjuvant chemotherapy, respectively. Although the patient showed a good histologic response to neoadjuvant chemotherapy, pulmonary metastasis developed 16 months after surgery

**Fig. 4:**
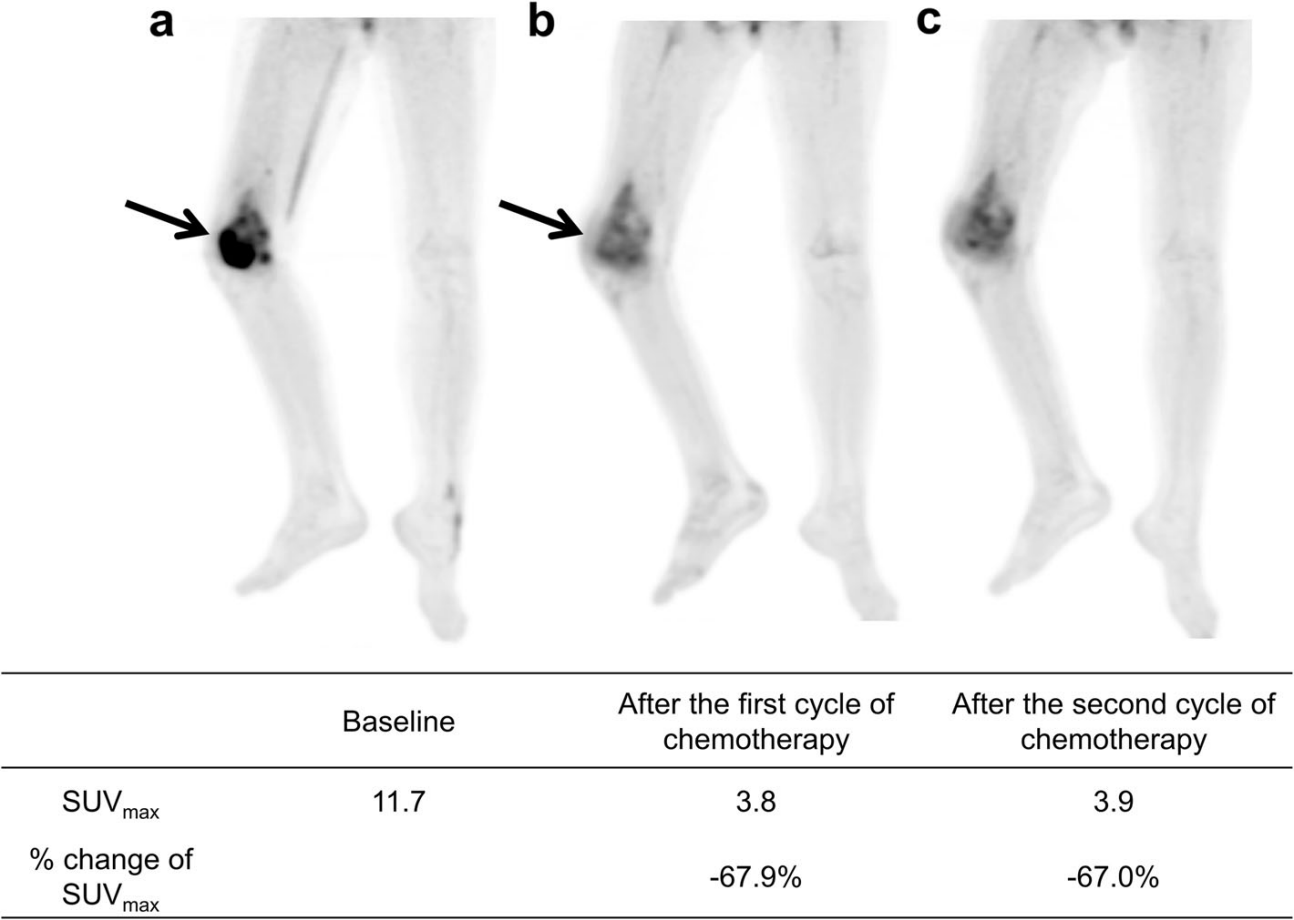
Maximum intensity projection images of [^18^F]FDG PET on the baseline (**a**), after the first cycle of neoadjuvant chemotherapy (**b**), and after the second cycles of neoadjuvant chemotherapy (**c**) of a 12-year-old female with osteosarcoma of the right femur. After neoadjuvant chemotherapy, SUV_max_ has reduced by 67.9% and 67.0% after 1 cycle and 2 cycles of neoadjuvant chemotherapy, respectively. Although the patient showed a poor histologic response to neoadjuvant chemotherapy, the patient is alive without recurrence for 112 months after surgery

The examples of different prognosis according to Δ%SUV01 and Δ%SUV02 are shown in Figs. 3 and 4.

## Discussion

In the current study, we evaluated the SUV_max_, a representative parameter of [^18^F]FDG PET/CT that can predict EFS of osteosarcoma.

Among the SUV_max_ measured at various time points during treatment, SUV2, Δ%SUV01, and Δ%SUV02 were found to be significantly associated with EFS. It is similar to previous findings [11]. However, it is not consistent with other studies that suggest that baseline SUV_max_ predicts EFS [12–14]. These inconsistencies may be due to the different populations evaluated in each study and the different follow-up time depending on each study.

Various studies have been conducted using [^18^F]FDG PET to predict the prognosis of osteosarcoma. The SUV_max_ of baseline [^18^F]FDG PET before NAC is related to the prognosis of osteosarcoma [12–14]. On the other hand, Sato et al. reported that baseline SUV_max_ is not correlated with EFS, but that SUV_max_ after completion of NAC is associated with EFS [11]. Furthermore, Im et al. reported that any SUV_max_ measured at baseline, interim, and posttherapy was not related to EFS [15]. Whereas, SUV_peak_, MTV, and TLG from baseline, interim, and post-therapy were related to EFS [15]. In our previous study, MTV at baseline was reported to have been associated with metastasis-free survival [9]. However, the values of MTV may be affected by the method selected for tumor delineation, which makes a consistent application to all studies difficult. Various [^18^F]FDG PET indices were reported to the EFS or metastasis-free survival (Additional file 1: Table S1). It was reported that several threshold methods for tumor delineation were used to evaluate MTV and TLG values [15], which means that it is difficult to establish an objective indicator with MTV and TLG for predicting prognosis.

All patients with high-grade osteosarcoma should undergo NAC according to the current NCCN guideline [16]. Osteosarcoma was one of the first solid tumors for which neoadjuvant chemotherapy proved to be beneficial [17, 18]. However, delaying surgery has been currently recognized as a dogma of osteosarcoma treatment [2]. Considering that the proportion of patients that show a good histologic response after NAC is about 50%, an increasing interest in up-front surgery instead of NAC is quite understandable [19–21]. Smeland et al. reported that the good histologic response to NAC was 52% [20]. Bielack et al. reported 56% of patients with osteosarcoma showed a good response to NAC [21]. Several investigators also showed less than 50% good responders with the presurgical chemotherapy. Meyers et al. reported 42% good responders and Kong et al. 44% [19, 22]. Currently, the histologic response to NAC is the most important prognostic criterion used to determine the treatment of localized osteosarcoma [18, 23, 24]. Previous studies have reported that [^18^F]FDG PET/CT can predict the histologic response of NAC in osteosarcoma [6, 25, 26]. However, recent studies raised a question about the true prognostic value of histologic response and required novel strategies for the treatment of osteosarcoma [27, 28]. Therefore, if the osteosarcoma patients’ prognosis can be evaluated early in the course of NAC, the timing of local control using surgical resection can be modified.

Our study suggested that SUV_max_ can be an important prognostic factor in osteosarcoma, which means that the early evaluation of treatment response of osteosarcoma is possible with the aid of [^18^F]FDG PET imaging. Although SUV0 and SUV1 themselves did not show significant correlations with events, Δ% SUV01 showed significant correlations with the predicting events. In other words, baseline and interim PET imaging during NAC can predict events early. This result could help change the treatment early, as it can select the patients with poor prognosis early. Davis et al. reported similar conclusion to ours [6]. Delayed tumor removal has several disadvantages and early surgery for non-responders may improve the clinical outcome of osteosarcoma. We believe that current stasis in survival improvement of osteosarcoma might be overcome with the early prediction of treatment with [^18^F]FDG PET.

In this study, the SUV_max_ was not analyzed as a continuous variable but was dichotomized with the cut-off values. It was already reported that the SUV_max_ as a continuous variable does not show statistical significance [15]. However, it is clear that the cut-off values from the analysis have its own limitation of being an arbitrary value which makes universal application difficult. Furthermore, another limitation is the difference between the cut-off values in different clinical studies. In order to apply [^18^F]FDG PET parameters into clinical use, many efforts to standardize the cut-off values will be required.

There are some limitations to our study. First, selection bias cannot be excluded due to the nature of the retrospective study. Second, there were only a small number of patients that were evaluated in this study. Third, the AUC values in the ROC curve analysis were not high. So, the accuracy of EFS prediction with SUV parameters was not high. This should be verified with a larger number of patients. Forth, factors affecting SUV include weight composition, patient weight, blood glucose level, and post-injection uptake time, etc., but SUV calculation basically includes correction of injection dose, uptake time, and body weight. However, body composition was not considered in this study. Adipose cells have significantly lower FDG uptake than other cells. Therefore, if the obesity level has changed, the possibility that SUV values of tumors can be measured differently cannot be excluded.

## Conclusion

PET evaluation after 1 cycle of presurgical chemotherapy predicted the clinical outcome of extremity osteosarcoma. [^18^F]FDG PET can be a useful modality for early response monitoring of neoadjuvant chemotherapy. Further evaluation with a prospective design will show whether early prediction of chemotherapy response can affect the modification of timing of local control. Adoption of this kind of personalized medicine will also help clinicians overcome the current stasis in survival improvement in osteosarcoma.

## Supplementary information

**Additional file 1: Table S1**. Summary of Previous Studies.

## Data Availability

The datasets used and/or analyzed during the current study are available from the corresponding author on reasonable request.

## Abbreviations

[^18^F]FDG: Fluorine 18-fluorodeoxyglucose
AJCC: American Joint Committee on Cancer
AUC: Area under the curve
CI: Confidence interval
EFS: Event-free survival
kVp: Kilovoltage peak
mA: Milliampere
MBq: Megabecquerel
MTV: Metabolic tumor volume
NAC: Neoadjuvant chemotherapy
NCCN: National Comprehensive Cancer Network
PET/CT: Positron emission tomography/computed tomography
PET0: PET/CT before neoadjuvant chemotherapy
PET1: PET/CT after one cycle of neoadjuvant chemotherapy
PET2: PET/CT after the completion of neoadjuvant chemotherapy
ROC: Receiver operating characteristic
SUV0: SUV_max_ on PET0
SUV1: SUV_max_ on PET1
SUV2: SUV_max_ on PET2
SUV_max_: Maximum standardized uptake value
SUV_mean_: Mean standardized uptake value
SUV_peak_: Peak standardized uptake value
TLG: Total lesion glycolysis
Δ%SUV01: Percentage change of SUV_max_ between PET0 and PET1
Δ%SUV02: Percentage change of SUV_max_ between PET0 and PET2
Δ%SUV12: Percentage change of SUV_max_ between PET1 and PET2

## References

[1] Vasquez L, Tarrillo F, Oscanoa M, Maza I, Geronimo J, Paredes G (2016). Analysis of prognostic factors in high-grade osteosarcoma of the extremities in children: A 15-Year Single-Institution Experience. Front Oncol.

[2] Harrison DJ, Geller DS, Gill JD, Lewis VO, Gorlick R (2018). Current and future therapeutic approaches for osteosarcoma. Expert Rev Anticancer Ther..

[3] Biermann JS, Adkins DR, Agulnik M, Benjamin RS, Brigman B, Butrynski JE (2013). Bone cancer. J Natl Compr Canc Netw..

[4] Geus-Oei LF, Oyen WJG. Predictive and prognostic value of FDG-PET. Cancer imaging. 2008;8:70–80. 10.1102/1470-7330.2008.0010 PMC2324370 18390390

[5] Song H, Jiao Y, Wei W, Ren X, Shen C, Qiu Z (2019). Can pretreatment ^18^F-FDG PET tumor texture features predict the outcomes of osteosarcoma treated by neoadjuvant chemotherapy?. Eur Radiol..

[6] Davis JC, Daw NC, Navid F, Billups CA, Wu J, Bahrami A (2018). ^18^F-FDG uptake during early adjuvant chemotherapy predicts histologic response in pediatric and young adult patients with osteosarcoma. J Nucl Med..

[7] Im H-J, Bradshaw T, Solaiyappan M, Cho SY (2018). Current methods to define metabolic tumor volume in positron emission tomography: which one is better?. Nucl Med Mol Imaging..

[8] Kong CB, Byun BH, Lim I, Choi CW, Lim SM, Song WS (2013). ^18^F-FDG PET SUVmax as an indicator of histopathologic response after neoadjuvant chemotherapy in extremity osteosarcoma. Eur J Nucl Med Mol Imaging..

[9] Byun BH, Kong CB, Park J, Seo Y, Lim I, Choi CW (2013). Initial metabolic tumor volume measured by ^18^F-FDG PET/CT can predict the outcome of osteosarcoma of the extremities. J Nucl Med..

[10] Durfee RA, Mohammed M, Luu HH (2016). Review of osteosarcoma and current management. Rheumatol Ther..

[11] Sato J, Yanagawa T, Dobashi Y, Yamaji T, Takagishi K, Watanabe H (2008). Prognostic significance of ^18^F-FDG uptake in primary osteosarcoma after but not before chemotherapy: a possible association with autocrine motility factor/phosphoglucose isomerase expression. Clin Exp Metastasis..

[12] Costelloe CM, Macapinlac HA, Madewell JE, Fitzgerald NE, Mawlawi OR, Rohren EM (2009). ^18^F-FDG PET/CT as an indicator of progression-free and overall survival in osteosarcoma. J Nucl Med..

[13] Franzius C, Bielack S, Flege S, Sciuk J, Jurgens H, Schober O (2002). Prognostic significance of ^18^F-FDG and ^99m^Tc-methylene diphosphonate uptake in primary osteosarcoma. J Nucl Med..

[14] Kubo T, Furuta T, Johan MP, Ochi M (2016). Prognostic significance of ^18^F-FDG PET at diagnosis in patients with soft tissue sarcoma and bone sarcoma; systematic review and meta-analysis. Eur J Cancer..

[15] Im HJ, Zhang Y, Wu H, Wu J, Daw NC, Navid F (2018). Prognostic value of metabolic and volumetric parameters of FDG PET in Pediatric Osteosarcoma: A Hypothesis-generating Study. Radiology..

[16] Biermann JS, Chow W, Reed DR, Lucas D, Adkins DR, Agulnik M (2017). NCCN Guidelines Insights: Bone Cancer, Version 2.2017. J Natl Compr Canc Netw.

[17] Whelan JS, Davis LE (2018). Osteosarcoma, Chondrosarcoma, and Chordoma. J Clin Oncol..

[18] Jeon DG, Song WS (2010). How can survival be improved in localized osteosarcoma?. Expert Rev Anticancer Ther..

[19] Kong CB, Song WS, Cho WH, Oh JM, Jeon DG (2012). Local recurrence has only a small effect on survival in high-risk extremity osteosarcoma. Clin Orthop Relat Res..

[20] Smeland S, Bielack SS, Whelan J, Bernstein M, Hogendoorn P, Krailo MD (2019). Survival and prognosis with osteosarcoma: outcomes in more than 2000 patients in the EURAMOS-1 (European and American Osteosarcoma Study) cohort. Eur J Cancer..

[21] Bielack SS, Kempf-Bielack B, Delling G, Exner GU, Flege S, Helmke K (2002). Prognostic factors in high-grade osteosarcoma of the extremities or trunk: an analysis of 1,702 patients treated on neoadjuvant cooperative osteosarcoma study group protocols. J Clin Oncol..

[22] Meyers PA, Heller G, Healey J, Huvos A, Lane J, Marcove R (1992). Chemotherapy for nonmetastatic osteogenic sarcoma: the Memorial Sloan-Kettering experience. J Clin Oncol..

[23] Bacci G, Longhi A, Fagioli F, Briccoli A, Versari M, Picci P (2005). Adjuvant and neoadjuvant chemotherapy for osteosarcoma of the extremities: 27 year experience at Rizzoli Institute. Italy. Eur J Cancer..

[24] Joo MW, Kang YK, Yoo CY, Cha SH, Chung YG (2017). Prognostic significance of chemotherapy-induced necrosis in osteosarcoma patients receiving pasteurized autografts. PLoS One..

[25] Harrison DJ, Parisi MT, Shulkin BL (2017). The Role of ^18^F-FDG-PET/CT in Pediatric Sarcoma. Semin Nucl Med..

[26] Lee I, Byun BH, Lim I, Kim BI, Kong CB, Song WS (2018). Comparison of ^99m^Tc-methyl diphosphonate bone scintigraphy and ^18^F-fluorodeoxyglucose positron emission tomography/computed tomography to predict histologic response to neoadjuvant chemotherapy in patients with osteosarcoma. Medicine..

[27] Bishop MW, Chang YC, Krailo MD, Meyers PA, Provisor AJ, Schwartz CL (2016). Assessing the prognostic significance of histologic response in osteosarcoma: a comparison of outcomes on CCG-782 and INT0133-A report from the Children’s Oncology Group Bone Tumor Committee. Pediatr Blood Cancer..

[28] Bishop MW, Janeway KA, Gorlick R (2016). Future directions in the treatment of osteosarcoma. Curr Opin Pediatr..

