# Supplementary material for: Early response monitoring of neoadjuvant chemotherapy using [18F]FDG PET can predict the clinical outcome of extremity osteosarcoma
Source: EJNMMI Res. 2020 Jan 3;10:1. doi: 10.1186/s13550-019-0588-4 (PMC6942108; doi:10.1186/s13550-019-0588-4)
Supplement: Supplementary file 1 — Additional file 1: Table S1. Summary of Previous Studies. [file 13550_2019_588_MOESM1_ESM.docx]

**Additional file 1**

**Table S1** Summary of Previous Studies

| Reference | Parameter | | Cut-off value | N | RR | 95% CI | P-value |
| --- | --- | --- | --- | --- | --- | --- | --- |
| Byun et al. | MTV (2.0) | Before CTx | ≤ 105 | 42 | 1 |  |  |
|  |  |  | > 105 | 41 | 3.93 | 1.55 - 9.92 | 0.004 |
| Costelloe et al. | SUV_max_ | Before CTx | < 15 | 27 | 1 |  |  |
|  |  |  | ≥ 15 | 4 | 4.51 | 1.34 -15.26 | 0.015 |
|  | SUV_max_ | After CTx | < 5 | 24 | 1 |  |  |
|  |  |  | ≥ 5 | 7 | 4.53 | 1.55 -13.24 | 0.006 |
| Im et al. | MTV (2.5) | Before CTx | < 238.06 | 21 | 1 |  |  |
|  |  |  | ≥ 238.06 | 13 | 5.02 | 1.51 -16.77 | 0.046 |
|  | MTV (2.5) | Interim | < 35.8 | 21 | 1 |  |  |
|  |  |  | ≥ 35.8 | 13 | 8.16 | 1.52 -43.69 | 0.046 |
|  | TLG (2.5) | Before CTx | < 981.97 | 20 | 1 |  |  |
|  |  |  | ≥ 981.97 | 14 | 5.74 | 1.34 -24.51 | 0.046 |
|  | TLG (2.5) | Interim | < 117.66 | 21 | 1 |  |  |
|  |  |  | ≥ 117.66 | 13 | 8.16 | 1.52 -43.69 | 0.046 |
|  | MTV (liver+2SD) | After CTx | < 2.2046 | 17 | 1 |  |  |
|  |  |  | ≥ 2.2049 | 17 | 11.77 | 1.57 -88.47 | 0.046 |
|  | TLG (liver+2SD) | Before CTx | < 1049.68952 | 19 | 1 |  |  |
|  |  |  | ≥ 1049.68952 | 15 | 4.43 | 1.33 -14.74 | 0.046 |
|  | TLG (liver+2SD) | After CTx | < 4.76543 | 15 | 1 |  |  |
|  |  |  | ≥ 4.76543 | 19 | 13.12 | 2.14 -80.57 | 0.046 |

RR, relative risk; CI, confidence interval; CTx, chemotherapy; MTV, metabolic tumor volume; TLG, total lesion glycolysis; SD, standard deviation.
